# Analysis of surgical and histopathological results of robot-assisted partial nephrectomy with use of three or four robotic arms: an early series results

**DOI:** 10.1590/S1677-5538.IBJU.2021.0495

**Published:** 2022-03-14

**Authors:** Lucas Schulze, Victor Teixeira Dubeux, José C. A. Milfont, Gustavo Peçanha, Pedro Ferrer, Andre Guilherme Cavalcanti

**Affiliations:** 1 Universidade Federal do Estado do Rio de Janeiro Departamento de Urologia Rio de Janeiro RJ Brasil Departamento de Urologia, Universidade Federal do Estado do Rio de Janeiro - UFRJ, Rio de Janeiro, RJ, Brasil; 2 Instituto de Urologia do Rio de Janeiro Rio de Janeiro RJ Brasil Instituto de Urologia do Rio de Janeiro, Rio de Janeiro, RJ, Brasil; 3 Universidade do Estado do Rio de Janeiro Centro Biomédico Rio de Janeiro RJ Brasil Centro Biomédico, Universidade do Estado do Rio de Janeiro - UERJ, Rio de Janeiro, RJ, Brasil; 4 Universidade Federal do Estado do Rio de Janeiro Disciplina de Urologia Rio de Janeiro RJ Brasil Disciplina de Urologia, Universidade Federal do Estado do Rio de Janeiro - UNIRIO, Rio de Janeiro, RJ, Brasil

**Keywords:** Kidney Neoplasms, Nephrectomy, Costs and Cost Analysis

## Abstract

**Objectives:**

The aim of this study was to evaluate whether criteria exist to guide election between the use the three- or four-arm technique in robotic partial nephrectomy (RPN) instead of just the surgeon’s preference.

**Material and Methods:**

We performed a retrospective review of 80 patients submitted to RPN from May 2016 to February 2020. The patients were divided into two groups of 40, the first submitted to the surgical procedure with use of three robotic arms and the second with four arms. The group division was performed independently of the complexity of the cases, age or gender of the patients and laterality of the renal lesions. Peri- and postoperative data were analyzed for comparison between the two groups.

**Results:**

Both techniques had similar oncological outcomes (positive tumor margins), renal function preservation (warm ischemia time) and hemorrhagic complications (estimated blood loss and renal artery pseudoaneurysm), with a small difference in the need for blood transfusion, favoring the technique with three arms.

**Conclusions:**

The two robotic partial nephrectomy techniques had similar oncological and postoperative outcomes, with minimal perioperative complications. The three-arm technique is safe and feasible regardless of the complexity and size of the tumor. Additionally, the use of the three-arm technique reduced surgery costs by US$ 413.00 per patient.

## INTRODUCTION

The early diagnosis of renal masses has increased in recent decades with advances of imaging technology (1). Approximately 60% of renal tumors are diagnosed in stage T1a (<4 cm). Recent reports have demonstrated the viability of performing partial nephrectomy in tumors at stages T1a and T1b (4-7 cm), making surgery the gold standard for treatment of small renal masses due to the possibility of oncological control with functional preservation and reduction of future cardiovascular risks (2, 3).

Since Gettman et al. described robotic partial nephrectomy (4), the procedure has spread significantly as an option for minimally invasive surgical treatment of small renal masses (5). The learning curve of minimally invasive partial nephrectomy can be reduced with the use of robotic platform as recently published in two series which showed that reasonable ‘Trifecta’ rates can be achieved even by low volume surgeons (6), and even with surgeons without previously laparoscopic experience (7). Robotic surgery offers all the benefits of minimally invasive procedures: shorter length of stay, less postoperative pain, reduced estimated blood loss and faster recovery (8).

The evolution of robotic surgical techniques has improved peri- and postoperative results of nephron-sparing surgery, such as reduction of warm ischemia time (9,10), lesser conversion into open or radical surgery (11), less severe postoperative complications (12) and better postoperative renal function (11).

There are many articles describing the outcomes of treating renal masses by robot-assisted surgery with use of three or four robotic arms, but the literature lacks studies specifically comparing the results of using three or four arms. Our objective was to evaluate whether criteria exist to determine the best technique to use, other than simple preference of the surgeon and if the three arms procedure is feasible for any case despite of tumor size, location and complexity. For this purpose, we sought to establish what parameters can be used for choosing between these two techniques by comparing the intra- and postoperative results of robot-assisted partial nephrectomy.

## MATERIALS AND METHODS

### Study design

This study retrospectively reviewed data from May 2016 to February 2020 of 80 patients submitted to robot-assisted partial nephrectomy. The procedures were performed by two surgeons with extensive experience in minimally invasive nephron-sparing surgery. The patients were divided into two groups: 40 consecutive three-arm RPN and 40 consecutive four-arm RPN were reviewed. The option between the two techniques was exclusively based on the surgeon’s preference. The group division was performed independently of the complexity of the cases, age or gender of the patients and laterality of the renal lesions. The study design was approved by the ethics committee of the Hospital Universitário Grafree Guinle (No 5258).

Preoperative demographic parameters and tumor characteristics were recorded. Intraoperative details such as operating console time, estimated blood loss, need for blood transfusion and warm ischemia time were recorded. Postoperative variables such as length of hospital stay, postoperative hemorrhage and need of angioembolization were reviewed. Four-arm utilization costs were calculated based on our institution’s contracted purchase price from Intuitive Surgical.

Two surgeons (J.M. and V.D.) with extensive experience in minimally invasive renal surgery performed all the surgical procedures. The Da Vinci-Si and Da Vinci-Xi systems (Intuitive Surgical, Sunnyvale, CA, USA) were used.

### Surgical technique

Following induction of general anesthesia, an orograstric tube and Foley catheter are placed. The patient is positioned in flank position with the affected side up. Mild table flexion may be applied to increase the space for ports. The ipsilateral arm is positioned to the side. Trocars are positioned after the pneumoperitoneum in direct view. Both techniques use only one 12 mm assistant port for suction, needle exchanges, Hem-o-Lock clipping and specimen bag deployment (see supplementary video).

After colon mobilization and retroperitoneum dissection with identification of the proximal ureter, the kidney is elevated by the fourth robotic arm to place the renal hilar vessels on stretch, enabling the two-handed dissection by the surgeon. When the procedure is performed with only three robotic arms, the assistant plays an important role in elevating the kidney and helping to expose the renal hilum.

The vascular clamping method was not randomized and was used according to the surgeon’s preference. Both techniques (three-arm or four-arm) were performed with transperitoneal access, and the renorrhaphy was performed by the sliding-clip technique (13) using V-Loc 3.0 sutures (Covidien). No hemostatic agent or double J catheter was employed.

### A - Four-arm technique ports placement

**Figure 1:**
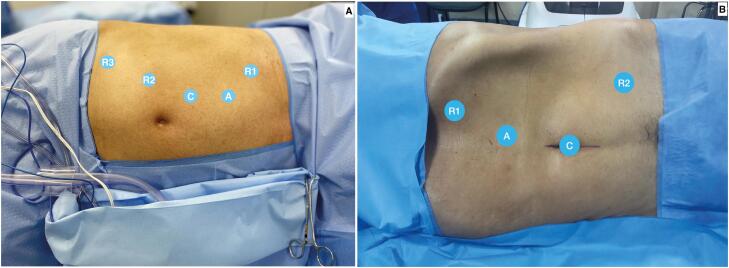
A) Four-arm port placement. Camera port placed medially to the umbilicus (C), midline assistant port (A), right working port (R1), left working port (R2) and third robotic arm (R3). B) Three-arm port placement. Camera port placed in the umbilicus (C), midline assistant port (A), left working port (R1) and right working port (R2).

The camera trocar is placed above the umbilical scar in the paramedian line. The pattern of port positioning is demonstrated in Figure-1A. The trocar angle can be adjusted slightly cranial or caudal according to tumor location. The 12 mm assistant port is placed cranially in the same line to the camera port at a minimum distance of 5 cm.

### B - Three-arm ports placement

The camera trocar is placed at the umbilicus to preserve abdominal aesthetics by using a natural scar. The pattern of port positioning is demonstrated in Figure-1B. The trocar angle can be adjusted slightly cranial or caudal according to tumor location. The 12 mm assistant port is placed cranially in the same line as the camera port at a minimum distance of 5 cm.

### Statistical analysis

The statistical analysis was carried out by cross-referencing the data of the two groups as a whole, as well as for each variable individually, to ascertain the possible impacts on the final result.

Initially, the data were used to calculate absolute frequencies and percentages (qualitative variables) and to compute descriptive statistics: mean, standard deviation, minimum, median and maximum (quantitative variables). To compare the groups regarding the qualitative variables, and thus to estimate the relative risks (RRs), Poisson regression with robust variance was used with the log-link function. For comparisons involving quantitative variables, linear regression with mixed effects (random and fixed effects) was used. These linear models with mixed effects are used to analyze data in which the responses are grouped (more than one measure for a single individual, since some participants underwent more than one operation), and when the assumption of independence between the observations in each group is not suitable. For comparisons, we used the orthogonal contrast post-test, while to compare the groups regarding the number of days in the hospital we also used Poisson regression with robust variance, but with the identity link function. All the comparisons were adjusted by the patient’s age, a possible confounding variable.

## RESULTS

A total of 80 patients underwent transperitoneal partial nephrectomy with warm renal ischemia. The patients’ demographic characteristics are reported in Table-1. Between the three-arm and four-arm groups, there were no differences in age, gender, tumor laterality, RENAL score nephrometry and tumor size. The average sizes of the tumors in the two groups were 3.82 cm (1.3-10.0) and 3.49 cm (1.0-8.5) for the procedures with three and four arms, respectively. The number of complex cases (RENAL score >10) were similar in both techniques, there were 12 cases (30%) in the three-arm group and 10 (25%) in the four-arm group. The average surgical time for three-arm technique was 81 minutes (29 – 215 minutes), while for the four-arm technique it was 91 minutes (40 – 180 minutes). The mean warm renal ischemia time of the three-arm group was 16.25 minutes (0 – 35 minutes) and of the four-arm group it was 21.78 minutes (0 – 50 minutes). There was no statistical difference in average surgical time between the two groups (RR 0.13 [CI -0.41 to 0.15], *p*= 0.23), as well as in the warm renal ischemia time (RR -5.15 [CI -11.3 to 1.00], *p*= 0.08). There was no statistical difference between the groups in the estimated perioperative blood loss (RR -0.05 [CI -0.67 to 0.57], *p*= 0.81). The average estimated blood loss in the three-arm group was 221 mL (30 – 800 mL), while in the four-arm group it was 325 mL (20 – 2,250 mL). There was a slight advantage in the comparative analysis regarding blood transfusion rate for the patients who underwent three-arm robotic surgery (RR 0.20 [CI 0.05 to 0.77], *p*= 0.02), a finding that might have been less evident with a larger sample size (Table-2).

**Table 1:** Patients distribution according to age, gender, tumor characteristics and histopathological findings.

VarIABLES		RPN 3 ARMS (n=40)	RPN 4 ARMS (n=40)	p VALUE
Age (years)		57.48 (20-75)	56.63 (34-77)	p=0.78
**Gender**				
	Male	23 (57.5%)	22 (55%)	p=0.82
	Female	17 (42.5%)	18 (45%)	
Diameter (cm)		3.82 (1.3-10)	3.49 (1.0-8.5)	p=0.42
**Renal Score**				
	4-6	19 (47.5%)	20 (50%)	p=0.88
	7-9	9 (22.5%)	10 (25%)	
	> 10	12 (30%)	10 (25%)	
**Laterality**				
	Left	22 (55%)	19 (47.5%)	p=0.50
	Right	18 (45%)	21 (52.5%)	
**Histopatology**				
	Angiomyolipoma	4 (10%)	4 (10%)	
	Oncocytoma	1 (2.5%)	2 (5%)	
	Benign cyst	3 (7.5%)	1 (2.5%)	
	Papiliferous	2 (5%)	3 (7.5%)	p=0.25
	Clear cells	29 (72.5%)	24 (60%)	
	Cystic nephroma	1 (2.5%)	0 (0%)	
	Chromophobe	0 (0%)	4 (10%)	
	Papillary clear cells	0 (0%)	2 (5%)	
**Stage**				
	Pt1a	32 (80%)	28 (70%)	
	Pt1b	2 (5%)	9 (22.5%)	p=0.10
	Pt2	5 (12.5%)	3 (7.5%)	
	Pt3	1 (2.5%)	0 (0%)	

**Table 2:** Per and post-operative data in patients` submitted to partial nephrectomy with three and four arms.

Variables		RPN 3 Arms (n=40)	RPN 4 Arms (n=40)	p Value
**Surgical Margins**				
	Negative	39 (97.5%)	39 (97.5%)	p=0.32
	Positive	1 (2.5%)	1 (2.5%)	
Estimated Blood Loss (mL)		221 (30-800)	325 (20-2,250)	p=0.81
Blood Transfusion		1 (2,5%)	3 (7.5%)	p=0.02
Console Time (minutes)		81 (29-215)	91 (40-180)	p=0.23
Warm Ischemia Time (minutes)		16.25 (0-35)	21.78 (0-50)	p=0.08
Renal Artery Pseudoaneurysm		1 (2.5%)	1(2.5%)	p=0.99

**RPN** = Robotic Partial Nephrectomy

Both groups presented the same number of positive surgical margins (one case), a rate of only 2.5%. The two groups also had the same number of hemorrhagic complications (renal artery pseudoaneurism), one in each group. Patients in both groups were submitted to embolization without need of any other surgical approach.

The ProGrasp® (Intuitive Surgical) grasper is commonly used in the fourth arm to dissect the kidney hilum and help during nephrorrhaphy. The unit cost of a ProGrasp® grasper is US$ 3,080.00, and this instrument can be used for 10 cases before expiring, so the per-case cost is US$ 308.00. The disposable canula seal costs US$ 25.20 and the drape utilized for the fourth arm costs US$ 21.00. The fourth arm’s sterile plastic cover costs US$ 58.80 per-procedure. The total amount saved with the three-arm technique is US$ 413.00 for each operation.

## DISCUSSION

Robotic partial nephrectomy became the preferred surgical technique by allowing treatment of complex renal masses with lower complication rates than traditional laparoscopy and open surgery (14). Both techniques applied to our sample produced similar oncological outcomes (positive surgical margins), renal function preservation (warm ischemia time) and hemorrhagic complications (estimated blood loss and renal artery pseudoaneurism). There was a small difference in the blood transfusion rate, favoring the technique with three robotic arms (RR 0.20 [CI 0.50-0.77], *p*=0.02).

The aesthetic aspects of the three-arms procedure previously described must be taken into consideration. When utilizing the umbilicus for the camera port and using only one 12 mm assistant port there is a significant reduction in postoperative abdominal scars. There was no technical difficulty to access kidney hilum or superior pole tumors by using the umbilical scar to perform a three-arm robotic partial nephrectomy, with the aesthetic benefit of using a natural body scar.

Although both methods are widely used, there are only a few studies comparing the outcomes of the three- and four-arm techniques. Recently, Johnson et al. (15) published a similar cohort study that demonstrated that robotic partial nephrectomy can be safely performed utilizing either 3 or 4 robotic arms, depending on surgeon preference, with slight differences in warm ischemia time and surgical margins between the two methods. We did not find any statistical difference when comparing margins and warm ischemia time between our two groups.

In recent years, the development of robotic surgery has made renal preservation safe and feasible in increasingly challenging cases (10). Moskowitz et al. reported high efficacy, better oncologic outcome, greater overall and cardiovascular survival rate among patients with small renal masses (T1a) when submitted to nephron-sparing surgery (16). In turn, Mir and collaborators conducted a systematic review and reported that partial nephrectomy can be performed safely with similar oncological outcomes in comparison with radical nephrectomy for the treatment of renal tumors up to 7 cm (17). Recent data also suggest that partial nephrectomy in tumors larger than 7 cm does not increase cancer-specific mortality (18, 19). In this study, the tumor diameter and the RENAL score status were not factors impeding performance of nephron-sparing surgery when technically feasible in both techniques. The average tumor diameter of the patients submitted to robotic surgery with three arms was 3.82 cm while the average size in the group that underwent surgery with four arms was 3.49 cm. Twelve (30%) patients undergoing the three arms procedures had a RENAL score greater than 10. The oncological outcomes were evaluated by the presence of positive surgical margins. There were only two cases, one in each group (2.5%), similar results to those recently reported (20).

Several studies have reported renal artery pseudoaneurysm as the most common and life-threatening complication following partial nephrectomy, regardless of surgical procedure. Scoll et al. described 110 patients that were submitted to robotic partial nephrectomy and showed postoperative blood transfusion rate of 3% and renal artery pseudoaneurysm requiring transfusion of 1% (21). This study’s rates of hemorrhagic complications with perioperative transfusion were 2.5% in the three-arm group and 7.5% in the four-arm group, while the rates of renal artery pseudoaneurysm requiring embolization were the same at 2.5%. The hemorrhagic complications were more often observed in this study in larger lesions (>4 cm), hilar renal masses and with collector system violation.

Renal function preservation is extremely important in evaluating the postoperative outcomes of patients submitted to nephron-sparing surgery (22). Recently published data report that warm ischemia time and quality and quantity of preserved kidney are the main factors determining postoperative renal function (23). Warm ischemia time under 25 minutes was the target in this study, which was achieved by 90% of the patients in both groups, with mean times of 16.25 minutes in the three-arm group and 21.78 minutes in the four-arm group. Furukawa and collaborators analyzed data on 130 patients and found that 81 presented warm ischemia time £ 25 minutes (mean of 21 minutes), with low perioperative complications, concluding that partial nephrectomy is safe and feasible even in the presence of hilar tumors (24). In our study, hilar and endophytic tumors (>50%) were associated with greater warm ischemia time but not associated with higher risk of hemorrhagic complications or impaired renal function.

Robotic surgery is more expensive than open and laparoscopic procedures (25). The total amount saved by not using the fourth arm was on average US$ 413.00 per procedure, due to the lower need for surgical supplies such as drapes, ProGrasp® forceps, canula seals and robotic arm sterile plastic covers. The cohort study of Johnson et al. (15) described savings of US$ 280.00 per procedure by avoiding the use of the same supplies. The savings reported in this study are even more significant, probably due to economic disparity between the two countries. This is highly relevant when choosing a surgical technique, by reducing hospital costs and allowing robot use in a larger number of patients. These economic aspects are particularly important in countries with low domestic technology, where universalization of access to robotic surgery depends on imported technology.

This study has several strengths. Both surgeons have extensive experience in minimally invasive renal procedures, with more than 20 years of clinical practice. All bedside assistants are certified robotic surgeons and no procedures were performed by residents or fellows. All patients in each group were treated by the same surgical team. There was no selection bias since a single surgeon was responsible for performing the procedures in each group regardless of tumor characteristics. The study’s major limitations are its retrospective nature and its small sample, which can lead to some analysis bias. Due to the complexity of some cases it’s is important to suggest that the three arms procedures should be performed by an experienced surgical team. Additionally, in cases of intraoperative necessity, the fourth arm is promptly available for use.

## CONCLUSION

The two robotic partial nephrectomy techniques evaluated had similar oncological and postoperative results, with minimal perioperative complications. The three-arm robot-assisted technique had a slight advantage regarding estimated blood loss and need for transfusion. There also was an important aesthetic benefit by utilizing the umbilicus for the camera port and using only one 12 mm assistant, with significant reduction in postoperative abdominal scars. Although cost was not the main objective of this study, the three-arm technique was substantially less expensive (US$ 413.00 per patient) due to the lesser cost of inputs, enabling greater use of robotic surgery.
